# Barley endosomal MONENSIN SENSITIVITY1 is a target of the powdery mildew effector CSEP0162 and plays a role in plant immunity

**DOI:** 10.1093/jxb/erac403

**Published:** 2022-10-13

**Authors:** Wenlin Liao, Mads E Nielsen, Carsten Pedersen, Wenjun Xie, Hans Thordal-Christensen

**Affiliations:** Department of Plant and Environmental Sciences, University of Copenhagen, Thorvaldsensvej 40, DK-1871 Frederiksberg C, Denmark; Department of Plant and Environmental Sciences, University of Copenhagen, Thorvaldsensvej 40, DK-1871 Frederiksberg C, Denmark; Department of Plant and Environmental Sciences, University of Copenhagen, Thorvaldsensvej 40, DK-1871 Frederiksberg C, Denmark; Department of Plant and Environmental Sciences, University of Copenhagen, Thorvaldsensvej 40, DK-1871 Frederiksberg C, Denmark; Department of Plant and Environmental Sciences, University of Copenhagen, Thorvaldsensvej 40, DK-1871 Frederiksberg C, Denmark; University of Ghent, Belgium

**Keywords:** Barley powdery mildew, *Blumeria hordei*, effector, encasement, *Hordeum vulgare*, immunity, MON1, multivesicular body, pathogen resistance

## Abstract

Encasements formed around haustoria and biotrophic hyphae as well as hypersensitive reaction (HR) cell death are essential plant immune responses to filamentous pathogens. In this study we examine the components that may contribute to the absence of these responses in susceptible barley attacked by the powdery mildew fungus. We find that the effector CSEP0162 from this pathogen targets plant MONENSIN SENSITIVITY1 (MON1), which is important for the fusion of multivesicular bodies to their target membranes. Overexpression of CSEP0162 and silencing of barley *MON1* both inhibit encasement formation. We find that the Arabidopsis ecotype No-0 has resistance to powdery mildew, and that this is partially dependent on MON1. Surprisingly, we find the MON1-dependent resistance in No-0 not only includes an encasement response, but also an effective HR. Similarly, silencing of *MON1* in barley also blocks *Mla3*-mediated HR-based powdery mildew resistance. Our results indicate that MON1 is a vital plant immunity component, and we speculate that the barley powdery mildew fungus introduces the effector CSEP0162 to target MON1 and hence reduce encasement formation and HR.

## Teaser

MON1 is essential for activation of Rab7-like GTPases and it is targeted by the barley powdery mildew effector CSEP0162, thus giving it an important role in plant immunity.

## Introduction

The plant immune system is activated in individual steps during the process of pathogen attack. Initially, plant plasma-membrane (PM) receptor kinases detect pathogen-associated molecular patterns and subsequently activate pattern-triggered immunity (PTI) (Zipfel and Oldroyd, 2017). As a countermeasure, the pathogens introduce effector molecules into the plant cell cytosol to prevent activation of PTI. However, these effectors may be recognized either directly or indirectly by plant nucleotide-binding leucine-rich repeat (NLR) receptors, whereby effector-triggered immunity (ETI) is activated, resulting in programmed cell death (Jones *et al.*, 2016; Kanja and Hammond-Kosack, 2020; Thordal-Christensen, 2020; Ngou *et al.*, 2022). PTI and ETI responses consist partly of a complex transcriptional reprogramming and partly of cellular responses. The latter include papillary cell wall appositions at sites of attack, encasements in the form of cell wall extensions that enclose the pathogen structures invading the plant cell, and the hypersensitive reaction (HR) programmed cell death. Papillae and encasements block penetration into the plant cell and nutrient transfer to the pathogen, respectively, while the HR is detrimental to biotrophic pathogens that depend on living plant cells.

From previous studies, several Arabidopsis proteins have been found that are important for penetration resistance towards the barley powdery mildew fungus (*Blumeria hordei*, *Bh*; formerly named *B. graminis* f.sp. *hordei*; Liu *et al.*, 2021), including the so-called PEN proteins (Hématy *et al.*, 2020). The syntaxin PEN1 (SYP121), as well as its barley orthologue ROR2, are required for timely papilla formation (Assaad *et al.*, 2004; Böhlenius *et al.*, 2010). PEN1 and its closest homologue, SYP122, have a shared function in papilla and encasement formation *per se* (Rubiato *et al.*, 2022). These syntaxins are probably required for the fusion of multivesicular bodies (MVBs) to the PM at the site of fungal attack in order to mediate papilla and encasement formation. Both these structures contain extracellular vesicles (EVs) that are secreted when MVBs fuse with the PM. These EVs are labelled with PEN1 (Nielsen *et al.*, 2012, 2017; Rutter and Innes, 2017), probably because this syntaxin is carried onto the intraluminal vesicles (ILVs) as they form in the MVBs. EV secretion into encasements, but not into papillae, is dependent on VPS9a, a Rab guanine-nucleotide exchange factor (GEF) that is required for activation of the Rab5 GTPases (Nielsen *et al.*, 2017). GTP-bound Rab5 GTPases are known to regulate the maturation of ESCRT-dependent MVBs and in addition they recruit the MONENSIN SENSITIVITY1/CALCIUM CAFFEINE ZINC SENSITIVITY1 (MON1/CCZ1) heterodimer that serves as a GEF to activate the Rab7 GTPases (Cui *et al.*, 2017). Completion of the Rab5→Rab7 transition is essential for the fusion of MVBs with the target membrane, which is either the tonoplast surrounding the vacuole, or alternatively the PM. Consequently, VPS9a is predicted to have a role in encasement formation as it recruits MON1/CCZ1 via activation of Rab5 to activate Rab7 (Hansen and Nielsen, 2018). The Arabidopsis Col-0 *MON1*-knockout (KO) mutants *mon1-1* (=*sand-1*) and *sand-2* have been found to suffer from very poor germination and impaired growth, whereas the *MON1*-KO mutant *mon1-2* of the Nossen-0 (No-0) ecotype is intermediate in size (Ebine *et al.*, 2014; Singh *et al.*, 2014; Cui *et al.*, 2017), confirming the fundamental importance of this gene in development. Interestingly, Ortmannová *et al.* (2022) recently studied Arabidopsis EXO70 complexes that are important for vesicle tethering to the PM, and they identified the EXO70B2 complex as interacting with PEN1 and to be required for normal papilla and encasement formation. Moreover, they also uncovered an interaction between EXO70B2 and RabG3C, an Arabidopsis Rab7 homologue, suggesting that PEN1 mediates a MVB–PM fusion that is regulated by the EXO70B2 complex during papilla and encasement formation.

Powdery mildew fungi are serious pathogens on numerous plant species, and their autonomous attacks on individual leaf epidermal cells make these plant–pathogen interactions useful for cellular studies. Powdery mildew fungi are biotrophic pathogens that take up nutrients from the plant via haustoria inside the host cells. The genome of *Bh* encodes hundreds of candidate secreted effector proteins (CSEPs) to promote attacks (Pedersen *et al.*, 2012; Frantzeskakis *et al.*, 2018); however, very few of these have been studied for their contribution to fungal virulence, let alone their target plant proteins. In the context of our current study, it is expected that some CSEPs hamper papilla and encasement formation in the barley host plant. In Arabidopsis, haustoria of the non-adapted *Bh* are generally encased (Nielsen *et al.*, 2017). In contrast, barley does not encase *Bh* haustoria, neither in compatible nor in incompatible interactions, even though the cellular machinery for making encasements exists, as demonstrated by the fact that this structure can be stimulated after treatment with the ergosterol biosynthesis-inhibiting fungicide, tetraconazole (Maffi *et al.*, 1995; Bolton *et al.*, 2016). Interestingly, haustoria developed from generative wheat powdery mildew hyphae are encased in wheat epidermal cells (Götz and Boyle, 1998). Therefore, we assume that *Bh* vegetative haustoria secrete CSEPs to effectively inhibit encasement formation.

CSEP0162 has previously been found to interact with small heat-shock proteins (sHSPs) (Ahmed *et al.*, 2015). In the current study, we used yeast two-hybrid analysis to re-screen for more barley proteins targeted by CSEP0162 and found that it also targets MON1. Overexpression of CSEP0162, as well as silencing of *MON1*, hampered encasement formation. Similar results were found in the loss-of-function mutant *mon1-2* of Arabidopsis, showing that MON1 serves a conserved function in encasement formation. More surprisingly, silencing of *MON1* was found to hamper barley NLR-mediated powdery mildew resistance. Similarly, *mon1-2* in Arabidopsis also hampered a cell death reaction induced by the powdery mildew fungus, suggesting that HR can be at least partially dependent on MON1. In support of MON1 also being a likely effector target in Arabidopsis, we provide evidence that the lethality of the Col-0 *mon1-1* mutant is partly due to EDS1-dependent autoimmunity.

## Materials and methods

### Plant material

The barley (*Hordeum vulgare*) lines used in this work were *ror2*, a syntaxin mutant in cv. Ingrid with low penetration resistance (Collins *et al.*, 2003), and the cv. Pallas near-isogenic lines P-01 and P-02, which have the powdery mildew resistance genes *Mla1* and *Mla3*, respectively (Kølster *et al.*, 1986). Barley plants were grown under a 16/8 h day/night photoperiod at 20/15 °C and 150 μmol m^−2^ s^−1^. The Arabidopsis lines used were the Columbia-0 (Col-0) ecotype and its mutants *mon1-1* (T-DNA insertion line SALK_075382; Singh *et al.*, 2014), *eds1-2* (Bartsch *et al.*, 2006), and *ndr1-1* (Century *et al.*, 1997), and the Nossen-0 (No-0) ecotype and its mutant *mon1-2* (Ds transposon line 54-4894-1 obtained from RIKEN, Japan) (Cui *et al.*, 2017). Arabidopsis plants were grown under an 8/16-h photoperiod at 21/15 °C and 125 μmol m^−2^ s^−1^. Arabidopsis germination rates were determined on half-strength MS phytoagar. Mutant allele genotypes were determined by PCR using the primers listed in Supplementary Table S1.

### Fungal material

The barley powdery mildew fungus (*Blumeria hordei*, *Bh*) isolates A6 and C15 were propagated on the cv. Pallas P-01 and P-02 lines, respectively, by weekly transfer. The Arabidopsis powdery mildew fungus *Golovinomyces orontii* (*Go*) isolate MPIPZ (*M*ax-*P*lanck-*I*nstitut für *P*flanzen*z*üchtungsforschung) was propagated on plants of Col-0 *eds1-2* by bi-weekly transfer.

### Construction of plasmids

The coding sequences (CDSs) of *HvMON1*, *HvMON1i*, and *CSEP0162* were amplified from the cDNA of *Bh*-inoculated barley (Ingrid) using Q5^®^ High-Fidelity DNA Polymerase (New England BioLabs, NEB) using the primers listed in Supplementary Table S1. The PCR products were cloned into the pDONR221 vector using BP Clonase (Invitrogen). Inserts were recombined into the destination vectors listed in Supplementary Table S2 by Gateway LR clonase (Invitrogen) reactions. All constructs were confirmed by sequencing.

### Yeast two-hybrid screening

For yeast two-hybrid analysis (Y2H), a barley cDNA library constructed from *Bh*-infected barley leaves and cloned into vector pDEST-ACT2 (Zhang *et al.*, 2012) was screened using the *Bh* effector pDEST-AS2-CSEP0162 bait-construct. The yeast (*Saccharomyces cerevisiae*) transformation protocol and recipe of synthetic dropout (SD) media, described by Zhang *et al.* (2012), were used to transform the prey library and the bait construct into yeast strains Y8800 and Y8930, respectively. These two haploid strains have different mating types and they have the mutations *ade2*, *his3*, *leu2*, and *trp1*. The strains were mated by mixing a 1 ml aliquot of the library strain and a 5-ml aliquot of the bait strain into 45 ml 2×YPDA medium in a 2 l flask, which was then incubated at 30 °C overnight with shaking at 30–50 rpm. The culture was then plated onto SD medium without Trp, Leu, His, and adenine, and with 2.5 mM 3-AT. After 2 d of incubation at 30 °C, the largest yeast colonies were picked for colony PCR and sequencing of the prey insert. Yeast colonies with prey constructs encoding barley proteins of more than 20 amino acids in-frame with the Gal4 activation domain were selected, and plasmids were extracted and retransformed into strain Y8800 to confirm the interaction. SNF1 (Y8930) and SNF4 (Y8800) were used as a positive control (Durfee *et al.*, 1993), and the negative control was pDEST-AS2-CSEP0105 (Ahmed *et al.*, 2015) and the empty vector.

### Bimolecular fluorescence complementation and protoplast protein co-localization

We used a set of Gateway binary Ti destination vectors with nGFP or cCFP and different fusion orientations, generated by Kamigaki *et al.* (2016). The full-length CDSs of *HvMON1* and *CSEP0162* (without signal peptide) were cloned into the bimolecular fluorescence complementation (BiFC) vectors by LR reactions and confirmed constructs were introduced into *Agrobacterium tumefaciens* strain GV3101. After incubation overnight, cultures were harvested and resuspended to OD_600_=0.7 in 10 mM MgCl_2_, 10 mM MES, and 0.1 mM Acetosyringone. A total of eight combinations of *A. tumefaciens* with constructs for *HvMON1* and *CSEP0162* fused to complementary nGFP/cCFP-fragments in different orientations (Supplementary Table S3) were co-infiltrated into *Nicotiana benthamiana* leaves. Dimerization of 14-3-3 was used as a positive control (Aitken, 2006).

Protoplasts were isolated from the second true leaves of 7-d-old barley according to Saur *et al.* (2019). The full-length CDSs of *HvMON1* and *CSEP0162* (without signal peptide) were cloned into the p35S-mCherry-GW and pUbi-GW-YFP vectors (Kwaaitaal *et al.*, 2010), respectively, by LR reactions and confirmed constructs were introduced and transformed into the protoplasts according to Saur *et al.* (2019).

The GFP signal in *N. benthamiana* 2 d after infiltration, and the mYFP and mCherry signals in protoplasts after overnight incubation in darkness, were detected using a Leica SP5 confocal microscope (GFP excitation at 488 nm, emission at 518-535 nm; mYFP excitation at 513 nm, emission at 526-555 nm; mCherry excitation at 588 nm, emission at 613–650 nm) at the Centre for Advanced Bioimaging, University of Copenhagen.

### Transient induced gene-silencing and overexpression in barley epidermal cells

Transiently induced gene-silenced (TIGS) of *HvMON1* was performed as described by Douchkov *et al.* (2005). A *HvMON1* RNA-interference (RNAi) fragment (316 bp) was designed using the siRNA-Finder (si-Fi) software (Lück *et al.*, 2019) and introduced twice in opposing orientations in the destination vector pIPKTA30N to produce a hairpin transcript (Douchkov *et al.*, 2005). A construct for overexpression of *YFP-CSEP0162* was previously generated by Ahmed *et al.* (2015). Each of these pIPKTA30N-*HvMON1i* and pUbi-*YFP*-*CSEP0162*-nos constructs were co-transformed with pUbi-*GUS*-nos as a marker into barley epidermal cells by particle bombardment according to Smigielski *et al.* (2019). The leaves were subsequently placed in closed 1% phytoagar plates with 10 μg ml^−1^ benzimidazole under a 16/8 h photoperiod at 20/15 °C and 150 μmol m^−2^ s^−1^. To study the transformed cells, the leaves were stained with X-Gluc for GUS activity according to Douchkov *et al.* (2005),

### Scoring of immune responses

To induce encasements around *Bh* haustoria, barley leaves were sprayed with 100 μg ml^−1^ tetraconazole in 20% acetone with 0.04% Tween-20 (Maffi *et al.*, 1995) 2 h before inoculation with *Bh*. To study the encasements, either alone or in combination with GUS staining, their callose content was visualized 5 d after inoculation after staining of the leaves with 0.01% Aniline Blue in 1 M glycine, pH 9.5, followed by UV epifluorescence microscopy.

Penetration rate, encasement formation, HR cells, and fungal development in Arabidopsis were scored by light and UV epifluorescence microscopy as described by Nielsen *et al.* (2017). Briefly, for scoring of penetration success, leaf material was stained with Trypan Blue according to Koch and Slusarenko (1990) 2 d after inoculation with *Bh* or *Go*. For each leaf, a minimum of 50 penetration attempts (presence of a fungal appressorium) were scored using light microscopy. Penetration was determined by the presence of a fungal haustorium. Callose staining was performed as described above.

### Quantification of gene expression by qRT-PCR and powdery mildew biomass by qPCR

Total RNA was extracted using the Monarch^®^ RNA Cleanup Kit (NEB). Reverse-transcription and cDNA synthesis were performed using the NEBNext^®^ RNA First Strand Synthesis Module (NEB). Transcript quantification was carried out using the Stratagene MX3000P real-time PCR detection system (Agilent Technologies) with FIREPol^®^ EvaGreen^®^ Mix (Solis BioDyne). The primers used to amplify PCR products of a maximum 200 bp are listed in Supplementary Table S1. The ubiquitin conjugating factor (*UBC2*) was used as the barley reference gene (Skov *et al.*, 2007). The level of gene expression was calculated using the relative quantification (2^–ΔΔ*C*T^) method (Livak and Schmittgen, 2001) by combining data from three separate experiments, each with two technical repeats.

Total genomic DNA of *Go*-inoculated Arabidopsis and *Bh*-inoculated barley was extracted using the DNeasy^®^ Plant Mini Kit (Qiagen). *Go* and *Bh* were quantified relative to plant DNA according to Weßling and Panstruga (2012) using the primers listed in Supplementary Table S1.

### Barley stripe mosaic virus-induced gene-silencing

The tripartite genome of barley stripe mosaic virus (BSMV) was used as the basis for virus-induced gene-silencing (VIGS) in barley. The binary Ti-constructs pCaBS-α, pCaBS-β, pCa-γbLIC, and pCaBS-γb-*TaPDS* have been described by Yuan *et al.* (2011). The Gateway cassette was inserted into the ligation-independent cloning site of pCa-γbLIC, and a RNAi fragment of *HvMON1* (the 316-bp fragment also used for TIGS) and the full-length CDS of *mYFP* were inserted by Gateway LR clonase (Invitrogen) reactions. All constructs were transformed into *A. tumefaciens* strain EHA105 by selection on rifampicin (25 μg ml^–1^) and kanamycin (100 μg ml^–1^). Confirmed strains were co-infiltrated into *N. benthamiana* with *A. tumefaciens* containing pCaBS-α and pCaBS-β according to the method described above. When BSMV symptoms appeared on upper leaves ~10 d post inoculation, infected leaves were collected and ground in 20 mM Na-phosphate, pH 7.2, with 1% silica. The homogenates were smeared onto the first true leaves of 7-day-old barley seedlings by rubbing gently with fingers. The third leaves of the treated barley plants were collected ~2 weeks later for either qRT-PCR or *Bh* inoculation. Silencing of phytoene desaturase using pCaBS-γb-*TaPDS* was used as a positive indicator for the VIGS system, while pCa-γb-mYFP was used as a negative control.

## Results

### CSEP0162 interacts with barley MON1

**Fig. 1.**
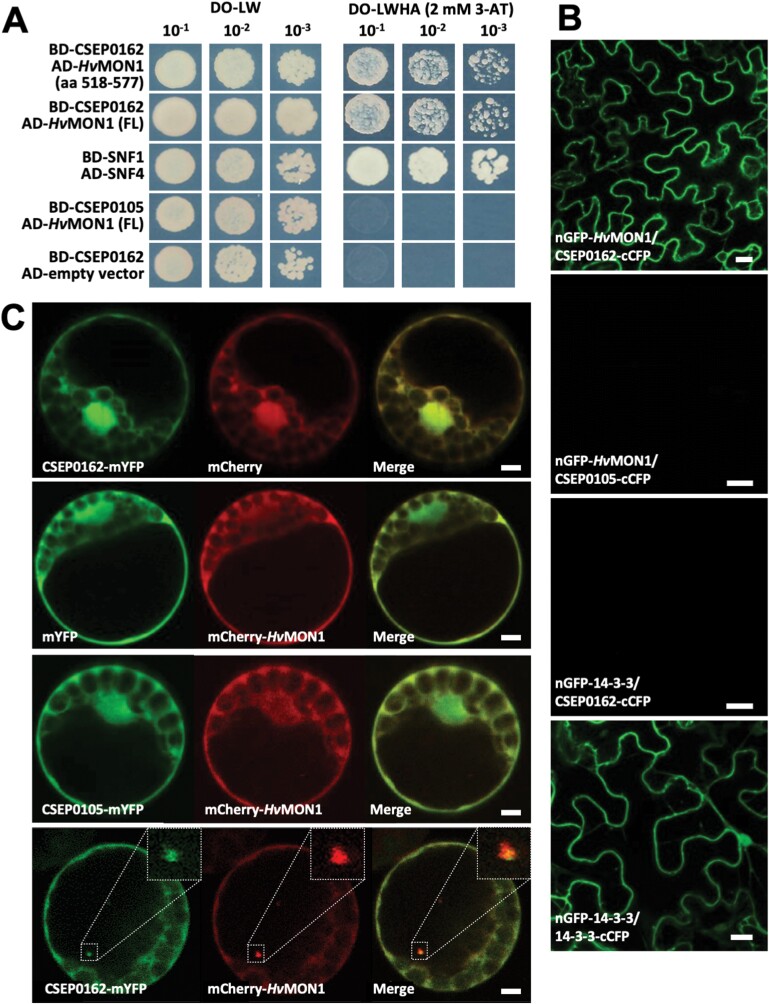
The *Blumeria hordei* effector CSEP0162 interacts with barley MON1 (HvMON1). (A) Yeast two-hybrid assays. Yeast transformed with constructs fusing CSEP0162 with the GAL4 binding-domain (BD) as well as the C-terminal and full-length (FL) HvMON1 with the activation-domain (AD). Growth on dropout (DO) medium lacking leucine (L) and tryptophan (W) indicates presence of both constructs. Growth on DO medium lacking L, W, histidine (H), and adenine (A) with 2 mM 3-amino-1,2,4-triazole (3-AT) indicates protein–protein interactions. SNF1/SNF4 was used as a positive control, CSEP0105 and the empty vector were used as negative controls. (B) Bimolecular fluorescence complementation in *N. benthamiana* leaves after *Agrobacterium* infiltration. Epidermal cells were observed by laser-scanning confocal microscopy (LSCM). The GFP signal was captured 48 h after co-expressing nGFP-HvMON1 and CSEP0162-cCFP. CSEP0105 and 14-3-3 were used as negative controls whilst dimerization of 14-3-3 protein was used as a positive control. Scale bars are 20 μm. (C) Co-expression of CSEP0162-mYFP and mCherry-*Hv*MON1 in barley P-02 protoplasts observed by LSCM at 24 h after transformation. Scale bars are 5 μm. The results were confirmed in at least three independent experiments.

The *Bh* effector candidate, CSEP0162, has previously been found to be expressed in haustoria, to contribute to fungal virulence, and to interact with sHSPs (Ahmed *et al.*, 2015). In a search for additional barley target proteins of CSEP0162, we re-screened the Y2H library used in Ahmed *et al.* (2015), which lead to the identification of a prey clone encoding the C-terminus of HvMON1 (amino acids 518–577). In support of this, the full-length HvMON1 was found also to interact with CSEP0162 in Y2H assays (Fig. 1A). The interaction between these two proteins occurred *in planta* as well, as shown by BiFC assays following agroinfiltration of *N. benthamiana* leaves, where the nGFP-HvMON1/CSEP0162-cCFP combination reconstituted a fluorescent protein, whereas the other seven combinations did not (Fig. 1B; Supplementary Table S3). CSEP0105 was used as negative control, as it was previously found also to interact with sHSPs (Ahmed *et al.*, 2015).

To study the interaction further, GFP-CSEP0162 and mCherry-HvMON1 were co-expressed in barley mesophyll protoplasts. As previously reported by Ahmed *et al.* (2015), GFP-CSEP0162 localized in the nucleus and cytoplasm, whereas mCherry-HvMON1 was only visible in the cytoplasm (Fig. 1C). Strikingly, CSEP0162 and HvMON1 co-localized in diffuse ~1 μm structures (insets in Fig. 1C; Supplementary Video S1). Similar structures were never observed when CSEP0162 and HvMON1 were expressed individually, or when HvMON1 was co-expressed with CSEP0105. Ahmed *et al.* (2015) observed somewhat larger diffuse structures when co-expressing CSEP0162 and interacting sHSPs, and referred to these as aggresomes. Indeed, sHSPs have the ability to enter aggresome formation together with interacting proteins (Johnston and Samant, 2021; Reinle *et al.*, 2022). Based on their similarity, we suggest that the CSEP0162/HvMON1 positive structures are also aggresomes, and we take this co-localization as additional evidence for molecular interaction between these proteins.

### HvMON1 and CSEP0162 regulate encasement formation around powdery mildew haustoria in barley

**Fig. 2.**
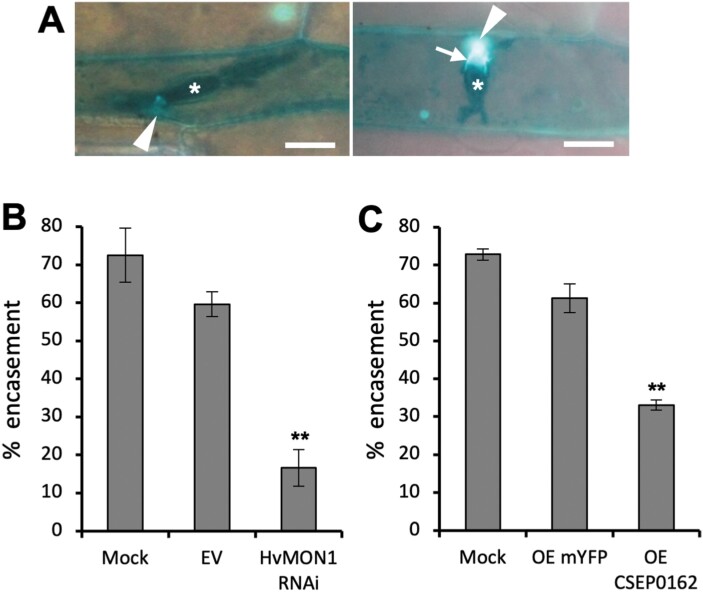
Encasement of *Blumeria hordei* (*Bh*) haustoria requires barley MON1 (HvMON1) and is suppressed by the effector CSEP0162. Leaves of 8-day-old barley plants were transformed by particle bombardment and 2 d later they were treated with tetraconazole and *Bh* inoculation. Encasement scorings were made after another 5 d. (A) *Bh* (C15 isolate) haustoria (asterisk) in epidermal cells of the barley *ror2* mutant with low penetration resistance without (left) and with callose-containing encasement (right, arrow), as imaged by UV-fluorescence microscopy after Aniline Blue treatment. Scale bars are 20 μm. (B) Effects of *MON1* RNAi and (C) CSEP0162 over-expression (OE) on encasement of the *Bh* haustoria in barley *ror2*. Mock, data from untransformed cells of leaves with *MON1*-RNAi and CSEP0162-OE cells, respectively. EV, empty vector; OE mYFP, transformed with the pUbi-GW-YFP construct. Data are means (±SE) of four experiments, each with scoring of at least 25 haustoria. Significant differences compared with the EV or OE-mYFP controls were determined using Student’s *t*-test: **P<0.01.

Previously, we have shown that VPS9a is required for the correct formation of encasements around powdery mildew haustoria in Arabidopsis (Nielsen *et al.*, 2017). MON1 acts downstream of VPS9a to activate Rab7, which in turn mediates MVB fusions to the tonoplast (Ebine *et al.*, 2014; Singh *et al.*, 2014; Cui *et al.*, 2017), and we hypothesized that this pathway would also mediate the MVB-to-PM fusion required for encasement formation. Therefore, we used RNAi-based TIGS to test whether HvMON1 is required for encasement formation around *Bh* haustoria in barley. We made use of tetraconazole to stimulate encasements in the barley epidermal cells (Maffi *et al.*, 1995; Supplementary Fig. S1) and found that TIGS of *HvMON1* reduced the formation of these defensive structures by more than 70% (Fig. 2A, B). Since CSEP0162 interacts with HvMON1, we speculated that this effector would also influence encasement formation. Therefore, we overexpressed CSEP0162 in the same set-up, and found a 50% reduction in encasement formation (Fig. 2C). Hence, our results suggested that CSEP0162 contributes to the inhibition of encasement formation by targeting HvMON1.

### MON1 is required for immunity in Arabidopsis

**Fig. 3.**
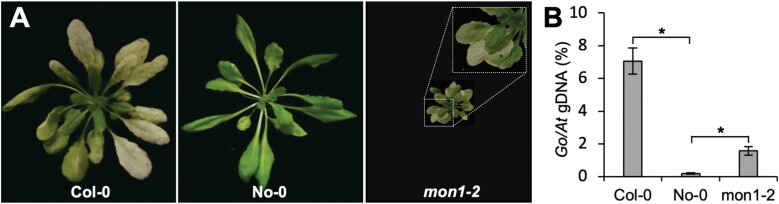
Resistance of the Arabidopsis No-0 ecotype to the powdery mildew *Golovinomyces orontii* (*Go*) requires AtMON1. (A) Plants of Col-0, No-0, and the No-0 *MON1*-knockout mutant *mon1-2* at 7 d after inoculation with *Go*. The resistance of No-0 is broken by the mutation. (B) qPCR-based quantification of fungal biomass at 6 d post inoculation. Data are means (±SE), *n*=3. Significant differences between means were determined using Student’s *t*-test: *P<0.05. The results were confirmed in at least three independent experiments.

MON1 is part of an evolutionarily conserved transport system that enables fusion of mature MVBs to their target membranes (Cui *et al.*, 2014; Ebine *et al.*, 2014; Singh *et al.*, 2014). Consistent with this, we found that overexpressing *GFP*-*HvMON1* in the Arabidopsis Col-0 *mon1-1* mutant reverted the phenotype to normal (Supplementary Fig. S2A, B). In the roots, the GFP-HvMON1 signal displayed a distinct punctate pattern, which in response to the PI3-kinase inhibitor wortmannin became ring-like, consistent with previous observations that AtMON1 localizes to the MVBs (Supplementary Fig. S2C; Singh *et al.*, 2014). Having confirmed that HvMON1 is the functional orthologue of Arabidopsis MON1, we next sought to determine whether MON1 has the same role in immunity in Arabidopsis to that observed in barley. To do this, we used the *mon1-2* mutant of the No-0 ecotype, which has superior growth relative to the Col-0 *mon1-1* mutant, including the fact that the homozygous No-0 *mon1-2* can grow to maturity and set seed (Cui *et al.*, 2017). Moreover, while seed germination is strongly impaired in Col-0 *mon1-1* (see below), the overall seed germination rate is unaffected in No-0 *mon1-2*. As with *Bh* on barley, *Go* is an example of a pathogen that has adapted to overcome the immunity presented by Arabidopsis, even in wild-type plants (Spanu *et al.*, 2010). However, in comparison to Col-0, we fortuitously discovered that No-0 is resistant to the *Go* powdery mildew fungal isolate MPIPZ. Strikingly, this resistance was to a large extent compromised in *mon1-2* (Fig. 3), showing that MON1 is indeed also essential for immunity in Arabidopsis.

### MON1 is required for normal penetration resistance, encasement formation, and HR in Arabidopsis

**Fig. 4.**
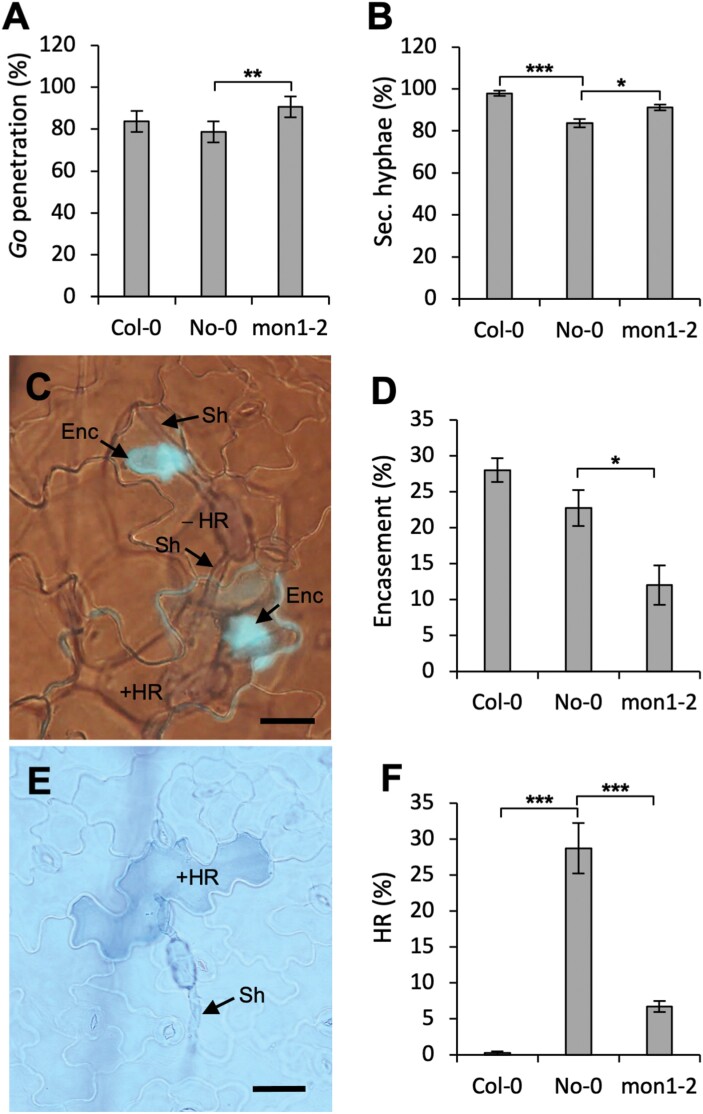
The MON1-dependent resistance of the Arabidopsis No-0 ecotype to the powdery mildew *Golovinomyces orontii* (*Go*) is manifested by encasement formation and a hypersensitive reaction (HR). (A) The percentage of fungal appressoria that successfully penetrated leaf epidermal cells of Col-0, No-0, and the No-0 *MON1*-knockout mutation *mon1-2*. (B) The percentage of successfully penetrating spores that had developed secondary hyphae in the three genotypes. (C) Representative image of No-0 leaf epidermal cells showing encasements around *Go* haustoria (Enc, stained blue), secondary hypha (Sh), and attacked cells with (+) and without (–) HR. (D) The frequency of encasements around *Go* haustoria in the three genotypes. (E) Representative image of HR of a single epidermal cell of No-0 attacked by *Go*. (F) Percentage of fungal appressoria-attacked cells showing the HR response in the three genotypes. All images and quantifications were obtained 2 d after inoculation. Scale bars are 50 μm. All data are means (±SE), *n*=4. Significant differences between means were determined using Student’s *t*-test: *P<0.05, **P<0.01, ***P<0.001. The results were confirmed in at least three independent experiments.

To further study the role of MON1 in the resistance displayed by No-0, we examined the initial stages of powdery mildew attack microscopically. We found that whilst *Go* had a high penetration rate in wild-type No-0, it was marginally increased in *mon1-2* (Fig. 4A). Furthermore, we found that the occurrence rate (Fig. 4B) and length (Supplementary Fig. S3) of secondary hyphae from successfully penetrating spores were increased on leaves of *mon1-2* compared to No-0, indicating that the post-invasive immunity of the mutant was hampered. Both Col-0 and No-0 displayed a significant encasement response to haustoria, which interestingly was reduced by ~50% in No-0 *mon1-2* (Fig. 4C, D). While this reduced encasement formation in part explains the increase of the growth rates of the secondary hyphae in *mon1-2*, it also shows that encasement formation in both monocots (Fig. 2B) and dicots (Fig. 4D) has a common requirement for MON1. In addition, while essentially none of the attacked cells in Col-0 underwent HR, this number was almost 30% in wild-type No-0. Remarkably, the number of cells that underwent HR in *mon1-2* was 75% lower than in No-0 (Fig. 4E, F). Taken together, this shows that MON1 is crucial for both the encasement and HR cell death immune responses. The effect of the *mon1-2* mutation on penetration resistance (Fig. 4A) might possibly be explained by a contribution of the encasement formation pathway to this pre-invasive immunity (see Discussion). Our results suggested that the powdery mildew resistance in No-0 is HR-mediated since penetration resistance and encasement formation were indistinguishable between No-0 and Col-0.

### HvMON1 is required for barley Mla3-mediated resistance to powdery mildew

**Fig. 5.**
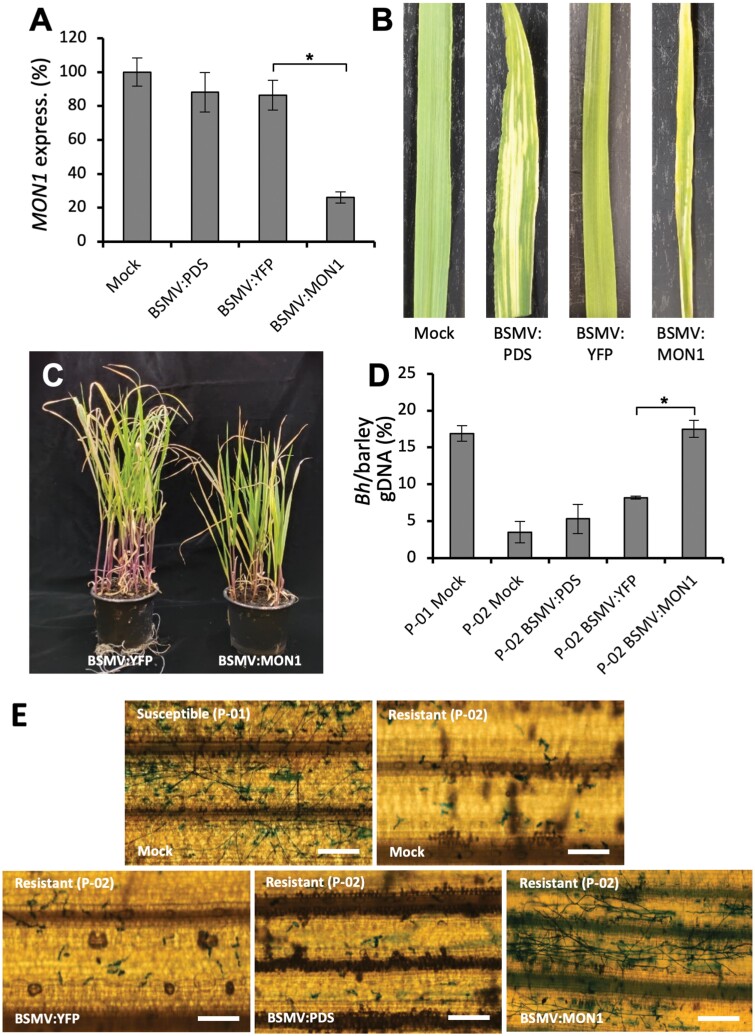
*Mla3*-mediated resistance to *Blumeria hordei* (*Bh*) in barley requires MON1. (A–C) Virus-induced gene-silencing (VIGS) using Barley stripe mosaic virus (BSMV) was employed to knockdown expression of *HvMON1*. Samples were taken from third leaves 14 d after virus inoculation of first leaves. Bleaching after VIGS-based knockdown of phytoene desaturase (*PDS*) and activation of RNA-silencing of *MON1* using the *YFP* coding sequence served as controls. Mock, untreated controls. (A) Expression of *HvMON1* as determined by qPCR. Expression is relative to that of ubiquitin conjugating factor (*UBC2*), and the value in the mock was set as 100%. (B) Images of individual leaves and (C) images of plants of BSMV:YFP and BSMV:MON1. (D, E) VIGS using BSMV in the barley near-isogenic cv. Pallas line P-01 that is susceptible to *Bh* fungal isolate A6 and in P-02 that has *Mla3*-mediated resistance. (D) qPCR-based quantification of fungal biomass at 4 d post inoculation (dpi) using the *Bh* glyceraldehyde-3-phosphate dehydrogenase and barley UBC2 genomic sequences. (E) Representative images of leaves at 4 dpi. Scale bars are 200 μm. All data are means (±SE) of three independent experiments, each with two technical repeats. Significant differences between means were determined using Student’s *t*-test: *P<0.05.

Previously, disease resistance and HR mediated by the coiled-coil NLRs (CNLs) RPM1 and RPS2 have been found to be dependent on the MVB components AMSH3 and VPS4 in Arabidopsis and *N. benthamiana* (Schultz-Larsen *et al.*, 2018). Together with our finding that *Go* induced a MON1-dependent HR in No-0, this led us to examine whether HvMON1 affects an Mla-mediated resistance to *Bh* in barley, since these are CNL-type R-proteins (Seeholzer *et al.*, 2010). To do this, we employed virus-induced gene-silencing (VIGS) of *HvMON1* in the barley line P-02, which harbors the *Mla3* allele. The resulting plants had a 65% reduction in *MON1* transcript levels (Fig. 5A); they were slightly smaller than the controls (Fig. 5B, C) but were otherwise amenable for study. Interestingly, in the *HvMON1*-silenced plants the *Mla3*-mediated resistance was strongly suppressed, and the level of disease reached the level of that of susceptible plants without *Mla3* (Fig. 5D, E). Thus, *Mla3*-mediated resistance requires HvMON1, and this supports the importance of functioning MVBs in CNL-mediated resistance.

### Germination defects and lethality of the Col-0 *mon1-1* mutant are partly immunity-dependent

**Fig. 6.**
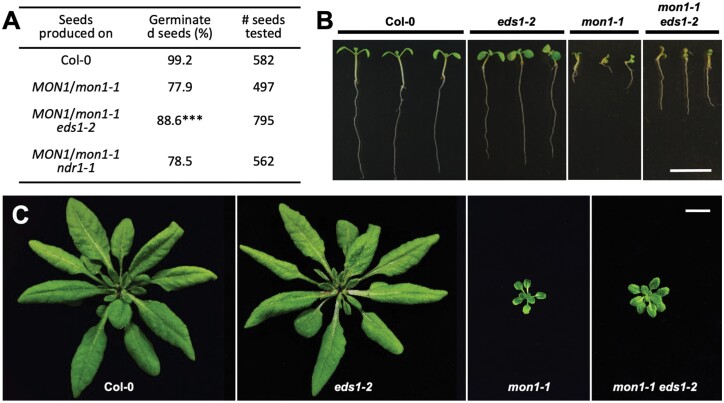
Lethality and reduced size of the Arabidopsis Col-0 *mon1-1* mutant is partly dependent on EDS1. The *eds1-2* and *ndr1-1* mutations were crossed into the Col-0 *MON1*/*mon1-1* heterozygous line, and then seeds of Col-0, Col-0 *MON1*/*mon1-1*, Col-0 *MON1*/*mon1-1 eds1-2*, and Col-0 *MON1*/*mon1-1 ndr1-1* plants were produced. (A) Germination rates of seeds segregating for *mon1-1* in the different genotypes. The significance of deviations from a seed germination rate of 75% of the Col-0 *MON1*/*mon1-1* lines was determined using Chi-squared tests. ***P<0.001. Development of (B) 10-day-old (plate-grown) and (C) 5-week-old (soil-grown) Col-0, *mon1-1* and *eds1-2* single-mutants, and *mon1-1 eds1-2* double-mutant. Scale bar is 1 cm. The plants were superior examples selected from those described in (A), and their genotypes were confirmed by PCR.

To counteract effectors removing or inactivating immune components vital for defense, NLRs often monitor potential targets of effectors either directly or indirectly. Consequently, the removal of such monitored immune components by mutations may activate NLRs inappropriately and cause severe developmental phenotypes (Thordal-Christensen, 2020). Given our finding that HvMON1 is an effector target of CSEP0162 and that MON1 plays a conserved role in plant immunity, we speculated that the severe lethality phenotypes described for the Col-0 *mon1-1* mutant could be a result of such a secondary activation of immunity (Cui *et al.*, 2017; Ebine *et al.*, 2014; Singh *et al.*, 2014). In Arabidopsis, most sensor NLRs are of the TIR-NLR (TNL) type, the rest being CNLs (Meyers *et al.*, 2003; Ngou *et al.*, 2022). While TNL immune activation always depends on EDS1, CNL immune activation can depend on NDR1 (Aarts *et al.*, 1998; Lapin *et al.*, 2019). We therefore crossed the *eds1-2* and *ndr1-1* mutations into the Col-0 *MON1*/*mon1-1* heterozygous line. Next, we produced seeds of Col-0, Col-0 *MON1*/*mon1-1*, Col-0 *MON1*/*mon1-1 eds1-2*, and Col-0 *MON1*/*mon1-1 ndr1-1* plants grown together under the same conditions and compared their germination rates. We found that 77.9% of the seeds of Col-0 *MON1*/*mon1-1* germinated, which was only marginally higher than the 75% that would be expected if all homozygous *mon1-1* seeds failed to germinate (Fig. 6A). This result was in good agreement with previous findings (Singh *et al.*, 2014). Moreover, the germination rate of Col-0 *MON1*/*mon1-1 ndr1-1* seeds was not different from that of Col-0 *MON1*/*mon1-1* seeds. Meanwhile, in the *eds1-2* background the Col-0 *MON1*/*mon1-1* seeds had a significantly higher germination rate (Fig. 6A). In addition, the improved germination was corroborated by the observation that *mon1-1 eds1-2* plants had longer roots and larger aerial parts than *mon1-1* plants (Fig. 6B, C). Overall, these results showed that the lethality inflicted by loss of MON1 was partly due to the activity of EDS1, which suggests that autoimmunity is activated by one or more TNLs.

## Discussion

In simple terms, papillae block penetration, whereas encasements are believed to prevent exchange of compounds between the haustorium and the plant cytosol, such as nutrients and possibly effectors. In barley attacked by *Bh*, encasement formation *per se* is not observed; however, early TEM studies observed a ‘collar’ formed around the neck of the haustorium as an extension from the papilla (e.g. Heitefuss and Ebrahim-Nesbat, 1986). We speculate that this is a rudiment of an encasement. In addition, Götz and Boyle (1998) found that haustoria developed from generative mycelium of the wheat powdery mildew fungus are encased in callose in wheat epidermal cells. Interestingly however, the collar/potential rudimentary encasement is very electron-light and distinct from the electron-dense papillae (Heitefuss and Ebrahim-Nesbat, 1986). The latter authors also described some papillae that are two-layered, with the first-formed layer being electron-dense and the layer formed later being electron-light. This electron-light layer is continuous with the collar. Nielsen *et al.* (2017) and Rubiato *et al.* (2022) have shown that in Arabidopsis attacked by *Bh* both the papillae and encasements are labelled with PEN1, while only the encasements are labelled with a constitutively active form of a Rab5 GTPase (ARA7^QL^) and SYP122. In the same studies, encasement formation was shown to be ARA7-dependent, whereas Böhlenius *et al.* (2010) and Nielsen *et al.* (2012) showed that papilla formation occurs via a separate pathway that is dependent on the ARF-GEF, GNOM, and ARFA1b/c GTPases. These observations would agree with a model saying that the electron-dense structure in the TEM study of Heitefuss and Ebrahim-Nesbat (1986) is the papilla and the electron-light structure is the encasement. This in turn would suggest that an encasement pathway is indeed activated in cereals attacked by powdery mildew, but that it is somehow hampered by the fungus. Furthermore, since this electron-light material is deposited as a layer onto the papilla, it might contribute to blocking penetration by *Bh*.

**Fig. 7.**
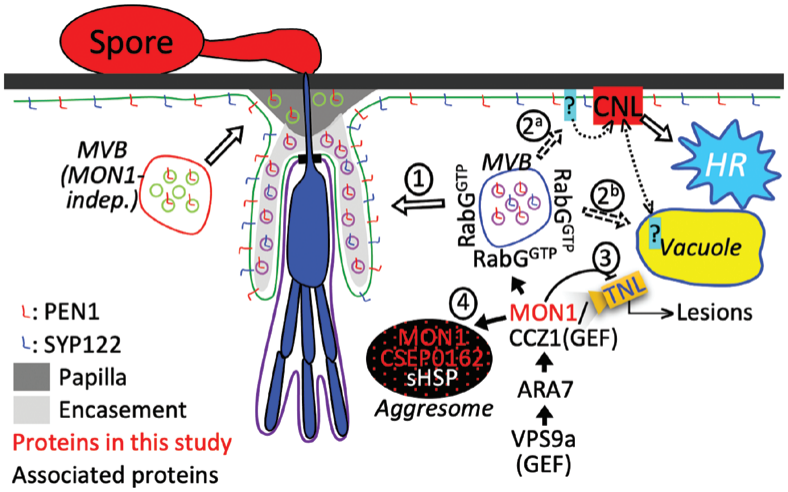
A model for the role of MON1 and the *Blumeria hordei* (*Bh*) effector CSEP0162 in immunity. MON1-activation of RabG associated with multivesicular bodies (MVB) contributes to immunity in several ways. (1) It allows the MVB to fuse with the plasma membrane (PM) for encasement formation. (2) It is required for coiled-coil nucleotide-binding leucine-rich repeat (CNL-)activated hypersensitive reaction (HR), as MVB fusion to the PM perhaps delivers a PM component required for CNL resistosome PM binding (2^a^), or MVB fusion to the vacuole sends a CNL negative regulator for degradation. When this degradation is prevented, the CNL is inhibited (2^b^). (3) In Arabidopsis, *MON1* knockout leads to EDS1-dependent developmental defects. Thus, MON1 is predicted to be directly or indirectly monitored by a TNL, which at the same time is inhibited by MON1. Upon MON1 inactivation/removal, this TNL is activated to cause plant lesions. (4) CSEP0162 interacts with MON1 and small heat-shock proteins (sHSPs). This might induce a MON1/CSEP0162/sHSP complex, observed as an aggresome-like structure, whereby MON1 is prevented from functioning at the MVB.

As a result of our current study, we can now add that MON1 is important for encasement formation (Figs 2B, 4D), and that the *Bh* effector CSEP0162 interacts with MON1 (Fig. 1) and at the same time inhibits encasement formation in barley (Fig. 2C). Previously, Ahmed *et al.* (2015) obtained a 40% reduction in the penetration rate of *Bh* by knockdown of CSEP0162 using host-induced gene-silencing (HIGS). The effect of CSEP0162 on encasement formation was not considered in that study; however, we speculate that the observed reduction in penetration might have been related to fortification of the papilla because of reduced CSEP0162-mediated inhibition of the encasement pathway (see model in Fig. 7). In Arabidopsis, encasements around *Bh* and *Go* haustoria are labelled with the PEN1 syntaxin, which is known to associate with extracellular vesicles (EVs; Nielsen *et al.*, 2017; Rutter and Innes, 2017). As the secretion of PEN1 into the encasement matrix depends on activation of ARA7 by its GEF, VPS9a, it suggests involvement of multivesicular bodies (MVBs) fusing to the plasma membrane (PM). Thus, MVB intraluminal vesicles labelled with PEN1 are secreted into the encasements as EVs, as suggested in the model in Fig. 7. Our present finding of MON1 being essential for encasement formation is very much in line with the previous findings referred to above, and it supports the idea that MVB fusion to the PM is required. We envisage that the MON1/CCZ1 complex is required to activate a RabG GTPase that might tether MVBs to the EXO70B2 complex, as suggested by Ortmannová *et al.* (2022). We also found that MON1 was important for reducing the penetration rate (Fig. 4), which also requires VPS9a and the EXO70B2 complex, as described by Nielsen *et al.* (2017) and Ortmannová *et al.* (2022). We believe this to be due to the encasement pathway fortifying the papilla by adding another layer onto it, as indicated by TEM study of Heitefuss and Ebrahim-Nesbat (1986).

The MON1-dependence of Mla3-mediated hypersensitive reaction (HR) described here (Fig. 5) together with previous findings that the MVB ESCRT components AMSH3 and SKD1 (VPS4) are required for resistance and HR activated by the CNLs RPM1 and RPS2 in Arabidopsis and *N. benthamiana* (Schultz-Larsen *et al.*, 2018) strongly suggests that MVBs serve a general function in CNL-activated HR. Therefore, according to the ‘iceberg model’ (Thordal-Christensen, 2020) it would not be surprising if the inhibition of MVB fusion with the PM or tonoplast inflicted by CSEP0162 could prevent other as yet unknown CNLs from activating HR. Conversely, silencing CSEP0162 by HIGS might stimulate HR of *Bh*-attacked cells and thereby reduce haustorium formation. As this was not addressed by Ahmed *et al.* (2015), future studies should test this hypothesis. Whether the MON1-dependent HR response against *Go* that we found in the Arabidopsis No-0 ecotype (Fig. 5) is activated by a CNL remains to be studied. In recent years, cell death activated by certain CNLs has been found to be caused by homopentameric CNL complexes, where the N-terminals of each CNL together form a pore in the plant cell PM, through which deleterious Ca^2+^ influx occurs (Wang *et al.*, 2019; Bi *et al.*, 2021). It will be interesting in the future to examine whether MVB traffic to the PM plays a role in this process (Fig. 7), as it has not escaped our attention that both the Mla10 N-terminus (identical to the Mla3 N-terminus; Seeholzer *et al.*, 2010) and the RPM1 N-terminus appear to be able to form such PM pores (Adachi *et al.*, 2019). Alternatively, CNLs might depend on MVBs to send a negative regulator into the vacuole for degradation.

Arabidopsis membrane-trafficking mutants often have growth defects due to loss of vital protein functions. Examples of this are *vps4* (*skd1*) (Haas *et al.*, 2007), *vps9a* (Goh *et al.*, 2007), and *gnom* (Mayer *et al.*, 1991). Nonetheless, our observation that the *eds1-2* mutation partially rescued the Col-0 *MON1*-KO mutant *mon1-1* (Fig. 6) suggests that autoimmunity adds to the severity of the phenotypic growth defect. Similarly, *eds1-2* also rescues *amsh3* mutants and therefore links autoimmunity with loss of normal MVB function (Schultz-Larsen *et al.*, 2018). It is becoming increasingly clear that immunity is often dependent on fundamental cellular processes such as membrane trafficking (e.g. Ortmannová *et al.*, 2022; Rubiato *et al.*, 2022), which are therefore potential effector targets. We also know that the NLR-based surveillance system monitors components targeted by pathogens and kills the cell when activated. Hence, we should expect some membrane-trafficking mutants to suffer from autoimmunity. Interestingly, the *MON1*-KO mutant *mon1-2* in No-0 performs significantly better than Col-0 *mon1-1* (Cui *et al.*, 2017). We speculate that this difference is due to the highly variable NLR populations present in the two ecotypes (Van de Weyer *et al.*, 2019).

One outstanding question is how CSEP0162 might suppress MON1 function. This effector has previously been shown to interact with two sHSPs (Ahmed *et al.*, 2015), and we found that CSEP0162 and MON1 co-localized in diffuse structures (Fig. 1C), which has not been observed when these two proteins have been expressed separately. We speculate these structures might be aggresomes similar to those formed when sHSPs interact with misfolded proteins (Johnston and Samant, 2021; Reinle *et al.*, 2022). Thus, we suggest that CSEP0162 links MON1 to sHSPs that subsequently induce formation of aggresomes containing all three proteins (Fig. 7). At an early stage of the interaction, sHSPs might attract larger HSPs to CSEP0162 and MON1, which targets them for ubiquitination and proteasomal degradation, or alternatively aggresomes might be removed by autophagy (Johnston and Samant, 2021; Reinle *et al.*, 2022), thereby preventing MON1 from functioning. A twist here is that MON1 itself is required for autophagy (Hegedüs *et al.*, 2016), suggesting that removal of the aggresomes in this case might be difficult.

In conclusion, our study shows that MON1 plays significant roles in encasement formation as well as in HR responses, and that the *Bh* effector CSEP0162 interacts with this protein, thereby making it important for immunity. We suggest that by this interaction and its additional interactions with sHSPs, CSEP0162 diverts MON1 into aggresomes and potential subsequent degradation. With such properties, we believe that MON1 and CSEP0162 are central to the interaction between barley and the powdery mildew fungus, and that this effector contributes to the fact that we see few encasements and little HR in compatible barley–*Bh* interactions.

## Supplementary data

The following supplementary data are available at *JXB* online.

Fig. S1. Callose-containing encasements around *Bh* haustoria in barley induced by tetraconazole.

Fig. S2. Barley MON1 complements the function of Arabidopsis MON1.

Fig. S3. Secondary hyphal length of *Go* in Arabidopsis No-0 and its *mon1-2* mutant.

Video S1. Co-expression of mYFP-CSEP0162 and mCherry-HvMON1 in barley P-02 protoplasts observed by laser scanning confocal microscopy at 20 h after transformation.

Table S1. Primers used in this work.

Table S2. Gateway destination vectors used in this work.

Table S3. Overview of HvMON1/CSEP0162 bifluorescence complementation results.

erac403_suppl_Supplementary_Figures_S1-S3_Tables_S1-S3

erac403_suppl_Supplementary_Video_S1

## Data Availability

The data that support the findings of this study are openly available at the Dryad Digital Repository. https://doi.org/10.5061/dryad.73n5tb30w; Liao *et al.* (2022).
