# Editor’s Note to: Barley endosomal MONENSIN SENSITIVITY1 is a target of the powdery mildew effector CSEP0162 and plays a role in plant immunity

**DOI:** 10.1093/jxb/eraf371

**Published:** 2025-11-17

This is an Editor’s Note to: Wenlin Liao, Mads E Nielsen, Carsten Pedersen, Wenjun Xie, Hans Thordal-Christensen, Barley endosomal MONENSIN SENSITIVITY1 is a target of the powdery mildew effector CSEP0162 and plays a role in plant immunity, *Journal of Experimental Botany*, Volume 74, Issue 1, 1 January 2023, Pages 118–129, https://doi.org/10.1093/jxb/erac403

In their continuing study of the interaction between the barley powdery mildew effector CSEP0162 and the barley MON1 protein the authors of this paper repeated the yeast 2-hybrid assay presented in Fig. 1A and found deviating results for the interaction between the full-length HvMON1 and CSEP0162. The authors present and discuss these new results in the following Insight article:

Deb S, Ar E, Liao W, Nielsen ME, Xie W, Pedersen C, Thordal-Christensen H. 2025. Barley powdery mildew effector CSEP0162 interacts with the endosome membrane-binding domain of barley MON1. *Journal of Experimental Botany*, Volume 76, Issue 20, 17 November 2025, Pages 5771–5774, https://doi.org/10.1093/jxb/eraf343

