# Supplementary material for: Barley endosomal MONENSIN SENSITIVITY1 is a target of the powdery mildew effector CSEP0162 and plays a role in plant immunity
Source: J Exp Bot. 2022 Oct 13;74(1):118–29. doi: 10.1093/jxb/erac403 (PMC9786837; doi:10.1093/jxb/erac403)
Supplement: erac403_suppl_Supplementary_Figures_S1-S3_Tables_S1-S3 [file erac403_suppl_supplementary_figures_s1-s3_tables_s1-s3.pdf]

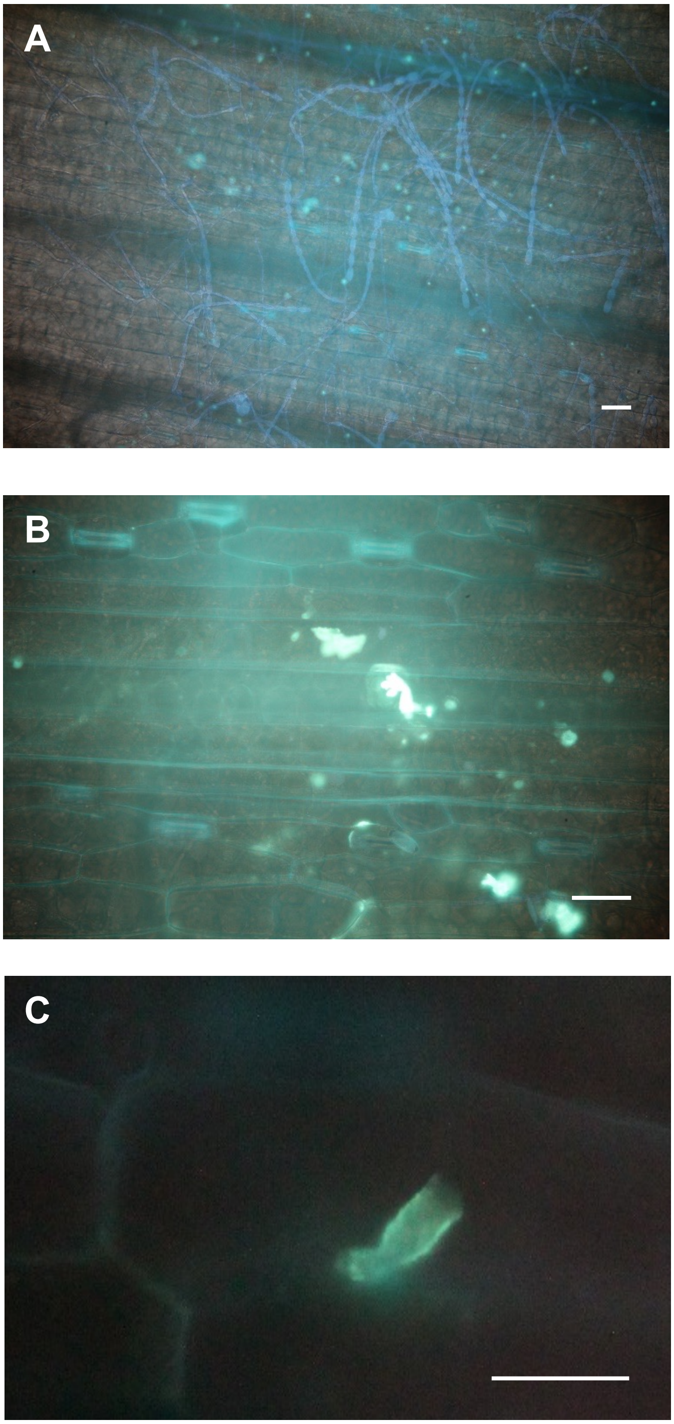

Supplementary Figure S1. Callose-containing encasements around *Bh* haustoria in barley induced by tetraconazole. (A) *Bh* colony and conidia chains developed on barley 4 days after inoculation. **(B,C)** Tetraconazole-induced encasements around *Bh* haustoria in barley epidermal cells. Inoculation was made 2 h after treatment. Images taken 4 dpi. UV-fluorescence microscopy after aniline blue treatment. Size bars, 100 µm in A and B, 10 µm in C.

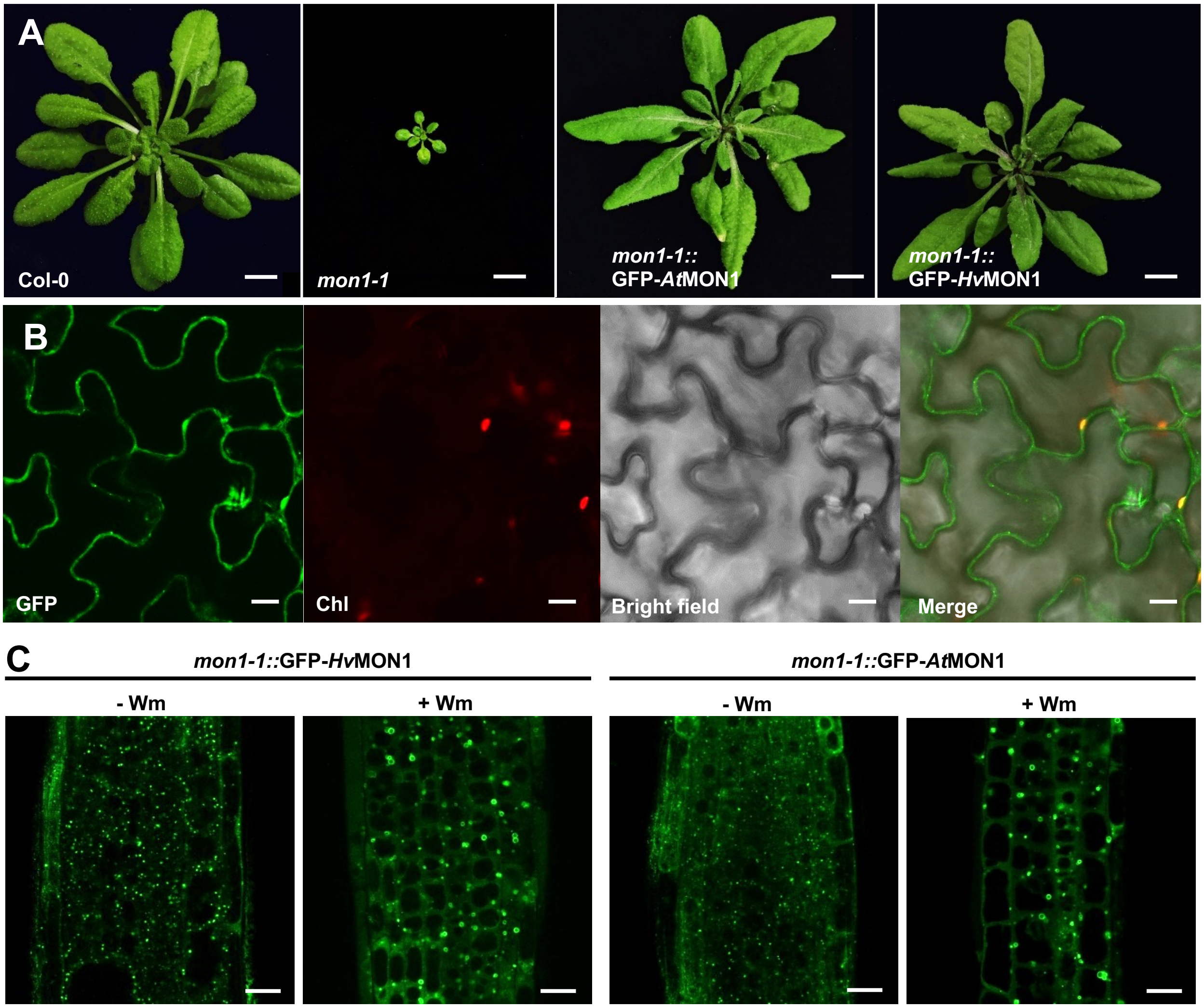

Supplementary Figure S2. Barley *Hv*MON1 complements the function of Arabidopsis *At*MON1. (A) Transformation of Col-0 *mon1-1* with *35S::*Hv*MON1* rescues it from its lethality phenotype. Size bars, 1 cm, **(B)** CSLM subcellular localization of GFP-*Hv*MON1 expressed in *mon1-1* leaf cells epidermal. **(C)** Wortmannin-induced ring-shaped structures of GFP-*Hv*MON1 and GFP-*At*MON1 resolved by CSLM. Roots were treated with 33 µM wortmannin for 1 hour. Size bars, 10 µm in B and 10 µm C.

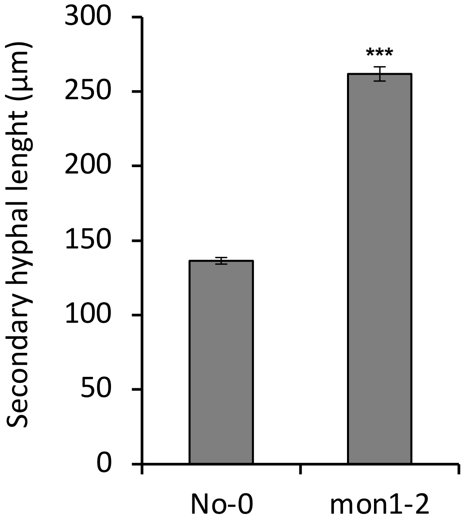

Supplementary Figure S3. Secondary hyphal lenght of *Go* on No-0 and *mon1-2.* Lenght of secondary hyphae developed from penetrated appressoria at 2 days after inoculation. Error bars, SE. ***, *P*<0.001 assessed by Student's T-tests. n=3.

Supplementary Table S1. Primers used in this work.

Name	Information	Sequence (5'-3')
**attbHvMON1-F**	Hv*MON1* Cloning	AAAAAAGCAGGCTACATGGATCCGGCCCCCGAT
**attbHvMON1-R**		AGAAAGCTGGGTCTCACCAGGCAATGGTGCT
**attBCSEP0162-F**	CSEP0162 Cloning	AAAAAGCAGGCTACATGGCCCAATATTCTAGACATATTAA
**attBCSEP0162-R**		AGAAAGCTGGGTCTCCCGAGCCAACTGCG
**attbAtMON1-F**	At*MON1* Cloning	AAAAAGCAGGCTACATGGCGACTTCAGATTCG
**attbAtMON1-R**		AGAAAGCTGGGTCTCACCAAGAGAAAGGACTAGCT
**pGB9-FP**	pDEST-AS2-1 sequencing	AGTGCGACATCATCATCG
**pGB9-RP**		CGTTTTAAAACCTAAGAGTCAC
**pACT2-FP**	pDEST-ACT2 sequencing	GATGATGAAGATACCCCAC
**pACT2-RP**		CAGTTGAAGTGAACTTGC
**attbHvMON1i-F**	Hv*MON1* RNAi Cloning	AAAAAGCAGGCTACCACCACTTCTAGGTGGCACA
**attbHvMON1i-R**		AGAAAGCTGGGTCGGCAAGCATATGGGTGAAAA
**qRT-VIGS-Mi-F1**	qRT-PCR for Hv*MON1* RNAi	CCGCACAAAACACAATTCAG
**qRT-VIGS-Mi-R1**		CCCTGATCCACTGGCATACT
**HvUBC2-F**		GGATCCAGGGCACCTCAC
**HvUBC2-R**		CGTCCAAGCTTTTTGAGGAC
**Bgh-GPD-F**	*Blumeria hordei* quantification	TGGCAATGCGTGTTCCTACT
**Bgh-GPD-R**		CATTTCCGGCGGCAATCTTT
**qPCR-HvM-F**		TTCTGATGAGGCAATTGGTG
**qPCR-HvM-R**		TTCTTATGGGCAAGGCAAAC
**BS10**	VIGS vector sequencing	GGTGCTTGATGCTTTGGATAAGG
**BS32**		TGGTCTTCCCTTGGGGGAC
**At3g21215-RBD-F**	*Golovinomyces orontii* quantification	GAATCCACCCATACCACCAG
**At3g21215-RBD-R**		GAGGAGGAGGATGGTGATGA
**GoATPase1-F**		TCGCCGCTATATTTGGAGTC
**GoATPase1-R**		CTGGGTCAGATGGTTCACCT
**mon1-mutant-LP**	mon1 mutant	CGGTTTGCCTGAGTTACTCAG
**mon1-mutant-RP**		AAAAGCCCAACAATATGGGTC
**DS5-3**	*mon1-2* (No-0)	TACCTCGGGTTCGAAATCGAT
**LBb1.3**	*mon1-1* (Col-0)	ATTTTGCCGATTTCGGAAC
**eds1-F760**	*eds1-2* mutant	ACACAAGGGTGATGCGAGACA
**eds1-R1458**		GGCTTGTATTCATCTTCTATCC
**eds1-R2333**		GTGGAAACCAAATTTGACATTAG
**ndr1-F3129**	*ndr1-1* mutant	GGTTGTGAAATCAAGAATTAATGTGGA
**ndr1-F4443**		TTGCCTAATGGATCGGCTG
**ndr1-R4928**		GTTCCTTGATTTGAAACCCAACA

Supplementary Table S2. Gateway destination vectors used in this work.

Name	Information	Reference
**pDEST-ACT2-GW**	Yeast two-hybrid	Robertson. 2004
**pDEST-AS2-GW**		
**pB4GWnG**	BiFC	Kamigaki et al. 2016
**pB4GWcCG**		
**pB4nGGW**		
**pB4cCGGW**		
**p35S-mCherry-GW**	Protoplast transformation and particle bombardment	Kwaaitaal et al. 2010
**pUbi-GW-YFP**		
**pUbi-GW-nos**		
**pIPKTA30N-GW**		Nowara et al. 2010
**pCaBS-γ-GW**	VIGS	pCaBS-γ (Yuan et al. 2011) were adapted to Gateway compatible
**pUBN10-GFP-GW**	Arabidopsis overexpression	Grefen et al. 2010

References

Grefen C, Donald N, Hashimoto K, Kudla J, Schumacher K, Blatt MR 2010). A ubiquitin-10 promoter-based vector set for fluorescent protein tagging facilitates temporal stability and native protein distribution in transient and stable expression studies. Plant Journal 64: 355–365

Kamigaki A, Nito K, Hikino K, Goto-Yamada S, Nishimura M, Nakagawa T, Mano S. 2016. Gateway vectors for simultaneous detection of multiple protein–protein interactions in plant cells using bimolecular fluorescence complementation. PLoS One **11,** e0160717.

Kwaaitaal M, Keinath NF, Pajonk S, Biskup C, Panstruga R. 2010. Combined bimolecular fluorescence complementation and Forster resonance energy transfer reveals ternary SNARE complex formation in living plant cells. Plant Physiology **152,** 1135–1147.

Nowara D, Gay A, Lacomme C, Shaw J, Ridout C, Douchkov D, Hensel G, Kumlehn J, Schweizer P. 2010. HIGS: host-induced gene silencing in the obligate biotrophic fungal pathogen Blumeria graminis. The Plant Cell **22,** 3130–3141.

Robertson M. 2004. Two transcription factors are negative regulators of gibberellin response in the HvSPY-signaling pathway in barley aleurone. Plant Physiology **136,** 2747–2761.

Yuan C, Li C, Yan L, Jackson AO, Liu Z, Han C, Yu J, Li D. 2011. A high throughput barley stripe mosaic virus vector for virus induced gene silencing in monocots and dicots. PLoS ONE **6**, e26468.

Supplementary Table S3. Overview of *Hv*MON1/CSEP0162 bifluorescence complementation (BiFC) results.

Combination	Fluorescence signal
*Hv*MON1-nGFP + CSEP0162-cCFP	No
*Hv*MON1-nGFP + cCFP-CSEP0162	No
nGFP-*Hv*MON1 + cCFP-CSEP0162	No
nGFP-*Hv*MON1 + CSEP0162-cCFP	Yes
*Hv*MON1-cCFP + CSEP0162-nGFP	No
*Hv*MON1-cCFP + nGFP-CSEP0162	No
cCFP-*Hv*MON1 + nGFP-CSEP0162	No
cCFP-*Hv*MON1 + CSEP0162-nGFP	No
